# Experienced vs. Novice Participants Perception of Overall Quality and Intention to Join in Future Sport Trials: Case European Duathlon Championship

**DOI:** 10.3390/ejihpe13080102

**Published:** 2023-07-28

**Authors:** Ana-María Magaz-González, César Sahelices-Pinto, Cristina Mendaña-Cuervo, Marta García-Tascón

**Affiliations:** 1 Departamento de Didáctica de la Expresión Musical, Plástica y Corporal y Educación Física, Facultad de Educación, Campus de Soria, Universidad de Valladolid, 42004 Valladolid, Spain; anamaria.magaz@uva.es; 2 Departamento de Dirección y Economía de la Empresa, Facultad de Ciencias Económicas y Empresariales, Campus de Vegazana, Universidad de León, 24071 León, Spain; 3 Departamento de Deporte e Informática, Facultad de Ciencias del Deporte, Universidad Pablo de Olavide, 41013 Seville, Spain; margata@upo.es

**Keywords:** perceived quality, general satisfaction, future intentions, athletes’ experience, PLS-SEM and fsQCA

## Abstract

Even though athletes’ experience has been ascertained to shape the perception of quality in sports events, scarce studies have addressed this issue jointly with the intent to join in upcoming comparable sports challenges. (1) Background: The present research investigates how the experience determines the evaluation of the championship and future intentions. (2) Methods: The PLS (SEM) method was evaluated specifically for both novice and experienced duathlon participants, and secondly, the fsQCA methodology was applied with the intention to estimate combinatorial net effects to confirm the hypothesis proposed. (3) Results reveal that the overall quality is equally important for novice and experienced individuals as a determinant of future intentions. However, novices tend to value more positively all the dimensions analyzed, while experienced ones noted higher levels of demand. (4) These findings highlight the importance of designing adequate management strategies for the participants with different levels of performance.

## 1. Introduction

Sports events represent a significant economic activity nowadays [1,2,3,4], especially in the tourism sector, and their popularity is used to promote cities, regions, and companies [5,6,7]. Even smaller-scale events have more potential for tourism development than mega-events [8,9].

As the sector becomes more competitive, the strife between cities and sports companies to host and organize sports events grows, while managers are aware of the need to differentiate sports events among the annual bid as well as to retain the participants’ loyalty for future editions since they are the real subjects. Participants are co-creators of these events [10,11], along with those who generate the sports competition, so their opinion must be considered by the organizers. In this sense, the perceived quality (PQ), the value it brings, and the satisfaction that a championship generates in athletes are key to building up the athletes’ loyalty and generating the future intention (FI) to repeat their participation in the championship, guaranteeing its continuity.

This represents the main reason why the analysis of the quality of sporting events is increasingly considered in related scientific studies. Research on the connection between perceived quality (PQ) and perceived value (PV), general satisfaction (GS), and loyalty has almost always been addressed from the point of view of the sports viewer [12,13,14,15,16], but fewer studies address it from the perspective of the athlete [13,17,18,19,20,21,22,23,24,25,26,27], who is also a co-creator of value and quality of the event with their participation [28,29]. These studies analyze quality in a static way without considering that the perception of the athlete may vary with experience and over time. Few studies explore repeated event participation and its effects on PQ, PV, GS, and sports event loyalty.

Based on this lack of research, this study is aimed at evaluating and comparing the impact of the sports experience on the PQ, in the overall quality (global quality -GQ-, GS, PV) of a sports event and, therefore, on future intentions (FI) through a double and innovative analysis in sport management: PLS-SEM and fsQCA.

## 2. Theoretical Background

The increase in the number of sporting events and their economic importance has long attracted researchers who analyze the keys to their success through the study of the PQ, the GS they generate, and the PV of participants and spectators to these types of events [12,13,19,30,31,32].

Quality perception is an opinion of the superiority of a service by comparing the expectations created about it with its actual performance [33]. Several instruments are used to try to explain it by considering different dimensions: SERVQUAL [34], a model by Rust and Oliver [35], and sub-dimensions [36,37,38,39]. Specific models are used in the field of sports management: SPORTSERV [40], EVENQUAL) [41], SEQSS [38], EPOD, and EPOD2 [42,43], which also introduce dimensions specific to each sporting event or service [25,27,44,45,46,47]. Furthermore, taking into account that perception may vary from spectators to participants [31], some analyses focus on spectators [14,44,48,49,50], and others on participants [13,17,30,51,52,53].

In addition, the perception of quality can be created at diverse stages of abstraction, considering both the broadest, such as overall service quality [54], as well as the most concrete, in relation to various sub-dimensions, or quality attributes, that make up the QP, as pointed out by Clemes et al. [37]. According to these authors, perceptions of different main dimensions influence the QP and QS. Magaz-González et al. [24], based on the Angosto-Sánchez questionnaire [55], Pérez-Campos [52], and Hightower et al. [14] evaluate QP based on dimensions and sub-dimensions, distinguishing between factors related to staff (S), communication (C), complementary services (CS), logistics (L), and duathlon specifics (DS).

On the other hand, the PV of an event by athletes has a double dimension, firstly, the usefulness and, secondly, the pleasure that such competition brings to them [56]. Moreover, the accumulated sports experience of the athletes must be considered [56], the co-creation of value with spectators [57], their tastes, preferences, needs, as well as the money, time, and effort invested [12,58,59]. Some authors evaluate PV from a multidimensional perspective with both positive and negative dimensions [60]. Perceiving values results in being more satisfied with the event, less sensitive to price, and an increasing willingness to repeat, so the literature indicates that PV is predictive not only of GS [15,61] but also of IF and loyalty [14,26,37,62,63].

Similarly, quality service generates general satisfaction (GS) in itself [35,36,64]. Satisfaction is the consumer’s overall assessment after a purchase [65], based on their consumption experience over time [66]. In sports services, where athletes are co-producers of the service [57] and have a high emotional involvement, satisfaction is also conditioned by emotional attachment [32,33,67,68] and by sporting achievements in competition [69]. It is, therefore, influenced by emotional and cognitive aspects [70], by experience, by athletic goal achievement [69], by the subjective perception of service [19,30], and by trust in the brand or organizing entity [71]. Research on this variable in sporting events uses various questionnaires to measure it and establishes that GS generates less price-sensitive clients [13,24,25,29,30,72] and encourages repeat participation [9]. The relationship between PV and GS has also been studied in sports environments. Most analysts argue that the PV affects GS [26,60,72,73,74,75], although some do not identify it [24,62].

Finally, sports event organizers want athletes to repeat their participation in successive editions. Knowing what motivates an athlete to participate again is important to them. For Zeithaml et al. [64], behavioral intent is a better predictor of actual behavior than PQ and GS. Others associate the effect of PV and GS, and thus indirectly PQ, with IF and the recommendation to attend the same or similar events [16,26,37,67,71,73,75,76,77,78,79,80,81,82] and reflect in their theoretical approaches, successive analyses of both direct and indirect impacts of QP, PV, and GS on IF. Other studies seek to understand the different profiles of participants in order to develop targeted marketing campaigns [69].

However, most of the studies cited above examine these interrelationships from a static point of view, without taking into account that the processes of evaluating a service change as it is experienced repeatedly, that consumers’ needs and evaluations change over time [83], people change their behavior [84] and, therefore, these relationships are not necessarily linear and static, but asymmetrical and dynamic [83]. From this perspective, little attention has been paid to how repeated participation in sporting events influences QP, PV, GS, as well as their direct and indirect effects on IF and how these effects are modified by the sporting experience.

In addition, the participant’s behavior changes over time as he or she considers the accumulation of information through experience. The trans-theoretical model of behavior (TTM) [84] is used to represent stages of change in attitudes, intentions, and behaviors, modifying habits, and has been applied to the field of physical activity practice on numerous occasions [85,86,87,88,89,90].

Using this model, the pre-contemplation stage can be equated to the moment when an athlete has little information about sporting events because it is the first time he or she has participated, influencing the perceptual factors of quality. In the next stage, contemplation, they can already consider other possibilities by having more to compare and confront the pros and cons of the event. In the preparation stage, with more experience, they seek information from several events to create an expectation of each one of them, analyze the factors that are most valuable for them to make the decision to act (participate). In the action stage, they have enough knowledge about the events to act, participating only in some of them. Finally, in the maintenance stage, their criteria for evaluating the quality of the event and their satisfaction with it are consolidated, and with all the experience accumulated, their intention to participate is totally defined. From this perspective, repeated participation in recurring events modifies their perception and shapes their behavior and IF.

Along the same lines, Avourdiadou and Theodorakis [83] collect marketing perspectives from which they study how experience influences the evaluation of a service, indicating that customers, over time, evaluate services differently because they have a greater amount of information relevant to the purchase, more experience and level of knowledge, so that as the consumer experience increases, more information and knowledge is acquired and the service is evaluated in a more complex way and with it PQ, GS, and loyalty. Therefore, this can be different for novice and experienced customers.

Kaplanidou and Gibson [9] equate past behavior with past participation in an event. This study also identifies past behavior at an event (participation) with the sporting experience at sporting events. In this sense, as these authors indicate, this experience can be a direct and positive predictor of intentions to re-consume the event.

Considering that there is little research with dynamic models in the literature on sports management, especially in sports events, and that the influence of experience in a championship on the evaluation modeling of a sport service has not been considered, the present study is aimed at examining the interactions between GQ, PV, and GS and their ability to predict participants’ IFs based on their experience, distinguishing between novice (N) and experienced (E) participants. Along these lines, the following general research hypotheses are proposed:

**H1:**  PQ positively influences the GQ of the event for both E (H1E) and N participants (H1N). This general hypothesis is subdivided into the following specific hypotheses:

**H1a:**  An adequate service by the organization’s staff (S) has a positive direct effect on the GQ of the event in E (H1aE) and N (H1aN).

**H1b:**  An effective communication (C) of the event has a positive direct effect on the GQ of the event in E (H1bE) and N (H1bN).

**H1c:**  Quality complementary services (CS) have a positive direct effect on the GQ of the event in E (H1cE) and N (H1cN).

**H1d:**  Efficient logistics (L) has a positive direct effect on the GQ of the event in E (H1dE) and N (H1dN).

**H1e:**  Proper management of the specific evidence elements (SD) has a positive direct effect on the GQ of the event in E (H1eE) and N (H1eN).

**H2:**  The GQ of the event has a positive direct effect on the PV of the event in E (H2E) and N (H2N).

**H3:**  The GQ of the event has a positive direct effect on the GS of the event in E (H3E) and N (H3N).

**H4:**  The PV of the event has a positive direct effect on the GS of the event in E (H4E) and N (H4N).

**H5:**  The PV of the event has a positive direct effect on the FI in E (H5E) and N (H5N).

**H6:**  The GS of the event has a positive direct effect on the FI in E (H6E) and N (H6N).

## 3. Materials and Methods

### 3.1. Sampling

**Figure 1:**
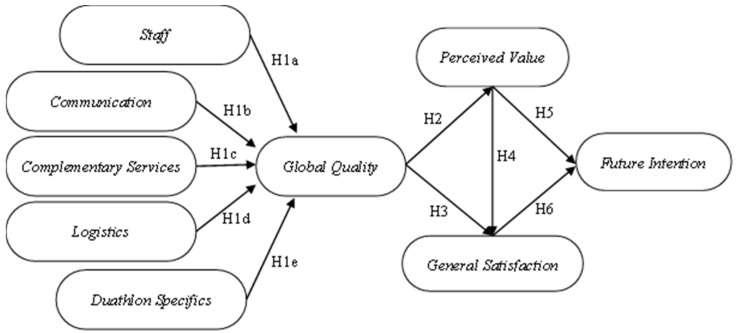
Estimation of the structural model.

To respond to the research hypotheses addressed above (see Figure 1), a questionnaire-based study was carried out during the European Duathlon Championship celebrated in the autonomous community of Castilla y León (Spain) in 2017. The duathlon is an individual athletic event similar to triathlons. The final sample comprised 210 athletes (N = 999; e = ±6.13%; α = 95.5), 151 of whom were men (71.9%) and 59 women (28.1%), whose ages ranged from 17 to 75 (μ = 41.16; σ = 14.22). Particularly, 112 of the 210 participants in the trial demonstrated an average of three or more years of competition experience in sporting challenges, such as the one mentioned above. The remaining participants, 98 people, reported less than three years of experience in events such as this (novice participants).

### 3.2. Measures

The instrument used was adapted from the validated questionnaire of Agosto-Sánchez et al. [17], according to the objective of the study and the specific peculiarities of the event.

A group of 11 experts was selected in order to assess the content validity of the instrument [91,92,93], with 5 women and 6 men: Academics (three university professors with more than 10 years of experience) and professionals in the sports industry (four athletes with more than six years of experience in competition experience and four members of the organization of this event).

Particularly, they were requested to analyze the relevance, clarity, simplicity, and comprehensibility of each item in relation to the objective of the study. They presented suggestions about the potential deletion/modification of existing items and/or the inclusion of prospective ones. Specifically, the agreement of 80% of experts to add an item to the final version of the instrument was required at least [94].

Finally, five items were eliminated from the sociodemographic section, three related to the PQ and one to the GQ. In addition, four items referring to the specific duathlon test were added. Eventually, the wording of two items of the “staff” dimension was modified for better comprehension.

To assess the reliability of the instrument, we evaluated the internal consistency through three indicators: Cronbach’s alpha (α) [95], composite reliability (ρc), and average variance extracted (AVE) indexes [96]. In this respect, values were above the minimum requirements.

With regard to the evaluation of the convergent validity of the instrument, we verified the significance of the standardized loadings (λ) in the CFA, and the correlation of each one of the dimensions of the study with the rest [97,98]. It was also assured that the item communalities (λ2) exceeded the level of 0.25. In general terms, the proposed model reflects an acceptable convergent validity.

In relation to the discriminant validity, it was verified that the manifest variable correlations were higher with their associated latent variable than with any other latent variable [97]. In this case, as well, indexes showed satisfactory discriminant validity.

The final version of the instrument consisted of four sections. The first section was dedicated to collecting data on key sociodemographic aspects, such as gender, nationality, age, category, and mode of participation, years of experience in national and international competitions, etc. Likewise, the second section was integrated by diverse scales concerning the five components or sub-dimensions of PQ (staff -four items-, communication -four items-, complementary services -five items-, logistics -four items-, and specific aspects of duathlon -eight items-). The third section was devoted to the measurement of the overall quality of the event. This was a multidimensional instrument divided into four concepts, namely, GQ -four items-, PV -four items-, GS -four items-, and FI -five items-.

To collect the responses, a five-point Likert scale was used, ranging from 1 (strongly disagree) to 5 (strongly agree).

All participants were asked to voluntarily answer via computer-aided personal interview (CAPI and web CAWI). The students from the Faculty of Education of Soria were the volunteers to collect the responses. Two weeks before the event, they met with the principal investigator, who explained the content of each scale and instructed them in the data collection tool to perform a reliable and objective data collection and with concordance among the volunteers.

Ethical review and approval were not required for the study on human participants in accordance with the local legislation and institutional requirements. Written informed consent to participate in this study was provided by all participants.

### 3.3. Statistical Procedure

Dimensionality properties and convergent and discriminant validities were evaluated specifically for both novice and experienced duathlon participants by means of the PLS Structural Equation Modeling (SEM) method deployed in the SmartPLS 2.0 software [99]. Moreover, data processing with PLS techniques explores and recreates a structure of optimal linear predictive paths with minimum requirements for the sample size [98,99,100,101].

Secondly, to confirm the previous analyses, the fsQCA methodology [102,103] was applied with the intention to estimate combinatorial net effects, not just independent net effects, whereby it heads to the identification of the possible conditions—both necessary and sufficient—conducting to a specific outcome. The objective was to carry out an analysis that allows us to know the relationships between the variables analyzed in the study (GQ, PV, and GS), and their ability to predict the FI and ~FI (non-FI) of the participants, considering the possible incidence of their experience in this type of events. Therefore, it was also considered appropriate to carry out the analysis for two different groups: Experimental and novice participants. Concretely, the application fsQCA 4.0 was used in this study [104].

## 4. Results

### 4.1. Structural Model: Experienced Participants

**Figure 2:**
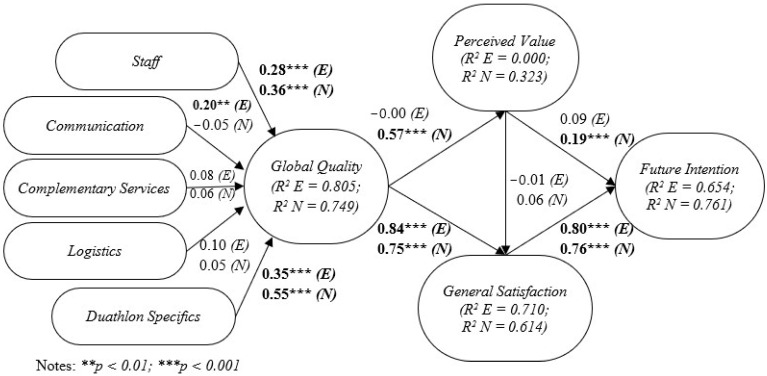
Structural model estimations for experienced (E) and novice (N) participants.

After the revision of the diverse procedures through which the reliability and validity of the measurement model were conveniently evaluated, the structural model was assessed initially for experienced participants and next, for novice participants using the PLS technique (Figure 2).

In this line, the scientific evidence showed that S (βS → GQ = 0.28, *p* < 0.001), C (βC → GQ = 0.20, *p* < 0.01), and DS (βDS → GQ = 0.35, *p* < 0.001) have a significative positive impact over GQ (Table 1), which indicates that hypotheses H1aE, H1bE, and H1eE are statistically supported. On the contrary, CS and L did not emerge as statistically significant. Their effect on GQ could not be proved, and hence, H1cE and H1dE were not supported.

For its part, PV came up as a disruptive dimension since none of the research hypotheses in which it is involved appeared to be significant (Table 1). In detail, GQ did not appear to have any sort of effect on PV (H2E not significant), nor PV on GS (H4E not significant), nor PV on FI (H5E not significant). Conversely, a positive effect of GQ on GS (βGQ → GS = 0.84, *p* < 0.001), and, in turn, GS on FI (βGS → FI = 0.80, *p* < 0.001) came out as significant. H3E and H6E were thus supported (Table 1).

Furthermore, Figure 2 also includes indexes of global adjustment of the structural model for experienced duathlon participants. According to Falk and Miller [105], the coefficient of determination (R^2^) obtained for each endogenous variable should be higher than 0.10. In this line, all latent constructs included in the structural model technically surpassed this threshold except for the case of PV (0.00). This fact highlights the poor explanatory and predictive capacity of PV over the hypotheses proposed in previous sections in reference to FI among duathlon participants with three or more years of experience in this sort of sports events.

### 4.2. Structural Model: Novice Participants

Continuedly, findings concerning the estimation of the structural model for the subgroup of novice duathlon participants are presented (Figure 2).

In this case, in reference to hypotheses H1aN and H1eN, these were supported since statistical evidence was found, demonstrating that S (βS → GQ = 0.36, *p* < 0.001) and DS (βDS → GQ = 0.55, *p* < 0.001) have a positive impact over GQ (Table 1). For their part, C, CS, and L did not seem to have any sort of effect on GQ. Hence, H1bN, H1cN, and H1dN were not supported.

Contrary to what occurred in respect of the experienced participants model, GQ appeared to have a significant effect on PV (βGQ → PV = 0.57, *p* < 0.001), and on GS (βGQ → GS = 0.75, *p* < 0.001), so hypotheses H2N, and H3N were supported.

On the contrary, whereas PV was confirmed to have a significant positive influence on FI (βPV → FI = 0.19, *p* < 0.001), the impact of PV on GS was not found relevant (H4N not significant). Only H5N was supported at this point (Table 1).

Finally, a positive effect of GS on FI (βGS → FI = 0.76, *p* < 0.001) came out as significant as well. H6N was thus supported (Table 1).

Moreover, Figure 2 shows the coefficient of determination (R^2^) obtained for each endogenous construct of the structural model for the case of novice participants. Once again, all latent variables exceeded by far the minimum required level of 0.10 (Figure 2).

### 4.3. Fuzzy-Set Qualitative Comparative Analysis (fsQCA): Experienced Participants

Considering all the findings obtained through the above approach, the following analysis aims to verify whether the combinations of variables in the model provide a more accurate explanation of IF, considering the user experience. In addition, it is also the objective of the research to know which combinations of conditions can elucidate the absence of IF to participate in sports events (~IF) depending on the profile of the participant.

In relation to the analysis of necessary conditions, in the case of both FI and ~FI, none of the consistency values in any of the conditions exceeds the threshold of 0.95, which is the most conservative assumption for this analysis. If this threshold is lowered, the GQ could be considered necessary because of the high consistency as well as its high coverage (in FI: Consistency 0.84, coverage 0.82; in ~FI: Consistency 0.83, coverage 0.85)

In the assessment of sufficient conditions, the intermediate solution has been chosen, further considering the proposal of Ragin [103] and Woodside [106], who suggest that a solution is remarkable if it reflects a consistency score above the threshold of 0.74.

As it can be seen in Table 2 (based on the notation used by Fiss [107]), there are two combinations of conditions (GQ; PV*GS) that explain 91% of IF, with a consistency of 0.807 (solution coverage: 0.91, solution consistency: 0.807). The configuration formed only by the GQ explains 84.31% of the cases, while the PV*GS configuration explains 64.35% of the analyzed options.

On the other hand, the same combinations of conditions in the negative direction (~GQ; ~PV*~GS) are given for the case of ~FI (solution coverage: 0.87, solution consistency: 0.82), which allows corroborating the conditions that must be given so that indeed an experienced athlete has the intention to go again to an event of these characteristics.

### 4.4. Fuzzy-Set Qualitative Comparative Analysis (fsQCA): Novice Participants

In the case of novice participants, none of the conditions can be considered necessary either (with the same threshold of 0.95), although if that parameter were lowered, GQ (presence and absence) could be considered necessary (in FI: consistency 0.87, coverage 0.83; in ~FI: consistency 0.82, coverage 0.86).

For this group (Table 2), there are three combinations of sufficiency conditions (GQ*PV; GQ*GS; PV*GS) that allow explaining 86% of FI, with a consistency of 0.848 (solution coverage: 0.858; solution consistency: 0.848). The most explanatory configuration is GQ*GS (explains 74.3% of cases), followed by the combination GQ*PV (which explains 70.7%), so it can be said that GQ continues to be one of the best explanatory combinations. Unlike the experienced, in this group, GQ alone is not sufficient. The combination PV*GS continues to be explanatory but with less coverage than in the case of the experienced, and a new combination of sufficient conditions (GQ*GS) appears.

However, in the case of ~FI, there are only two combinations of solutions (~GS; ~GQ) that can explain 91.3% of ~FI, with a consistency of 0.784. In this case, again, the absence of GQ is the most explanatory, so it can be concluded that it is indeed the most valued aspect both in the positive and negative sense, while the PV has no incidence.

## 5. Discussion

Participation in sports events has become a form of leisure and sports tourism with a great impact on the social, economic, and political levels. The organization of an event is a challenge for managers, who strive to develop differentiating strategies to ensure the continuity of the event. This paper investigates how the experience of participating in repeated events in PQ, GQ, PV, and GS impacts determining the FI of return in order to design strategies adapted to each niche of participants.

In general, at the methodological level, the results of the analysis show good levels of reliability and validity for the proposed dimensions, which empirically supports the suitability of the proposed measurement model.

In particular, the analysis of data using the PLS-SEM technique has shown that both novice and experienced athletes perceive the quality of the event, mainly through the performance of the personnel (S-GQ) and the elements of the duathlon (DS-GQ), confirming hypotheses H1a and H1e, and as observed in previous studies [14,32,38,40,49,50,108], displaying the importance of these factors and the convenience of considering specific dimensions of each event [25,109] and sub-dimensions to improve quality appreciation [37].

For both groups, S and DS have a positive influence on GQ and, therefore, on IF. According to hypotheses H1c and H1d, CS and L do not seem to play an important role in either scenario (N and E), are therefore not confirmed. In the same line, GQ also has a positive influence on GS (according to H3) and these, in turn, have a positive influence on IF (H6) for both novice and experienced participants, an influence also found by Kaplanidou and Gibson [9] or Avourdiadou and Theodorakis [83]. However, GS is not influenced by PV for any of the novice and experienced groups (according to H4—not confirmed).

Divergent aspects have also been found for each group. In the case of experienced participants (E), the C is shown to have a positive effect on the GQ (H1b -confirmed E-) and, in turn, on the IF, which is not the case for novice participants (H1b -not confirmed N-). On the other hand, GQ shows to have a positive influence on PV (H2) and this one on IF (according to H5) in the group of novice participants (N) that is not in the model estimated for experienced participants (E) (H2 and H5 not confirmed-E). In this sense, it can be stated that the level of experience does modulate the intention of future participation, in contrast to Kaplanidou and Gibson [9], who did not find that past experience with the event was not a predictor of the intention to participate again.

Moreover, the application of fsQCA has allowed the analysis of the interactions between different independent variables, an aspect that PLS-SEM does not allow. In general, although it seems that there are no necessary conditions in either group (N and E), sufficient combinations are obtained to understand the aspects affecting IFs of participating in sports events. It also makes it easier to understand the negative aspects that lead to ~FI in each group.

Thus, while in both groups GQ is the strongest condition (for both FI and ~FI), in the case of the experienced, it is a valid condition on its own for FI, while in the case of novices, no single condition is explanatory. However, in the case of the ~FI, both groups participate in the absence of GQ in an event, which affects their opinion on not intending to repeat participation. These results ratify the previous statement regarding GQ as the strongest condition, both in positive (FI) and negative (~FI). In relation to the possible combinations, both groups participate that the PV with GS affects favorably their IF.

In relation to the experienced group, in addition to GQ alone, the combination of PV and GS is explanatory of both FI and ~FI. In fact, in this group, the same combinations that explain FI, are explanatory of ~FI (in absence), which may be due to the fact that experience makes it easier to recognize the usefulness of the event compared to other aspects, such as price.

The results for FI in the case of novices imply three possible combinations, in none of which there is a single explanatory variable: There must always be a combination, either of GQ with PV (GQ*PV), of GQ with GS (GQ*GS) or of PV with GS (GS*PV). Furthermore, in this group, the explanation of their ~FI does not coincide with these combinations, but rather they are explained either by the absence of GS or by the absence of GQ (~GS; ~GQ). Unlike the experienced, in this group, no single conclusion can be drawn about their valuation since it is different depending on FI or ~FI.

In general, considering both techniques together (PLS-SEM and fsQCA), the findings show that GQ is a fundamental dimension in both segments and that it largely determines the intention to participate in sports events of this type in future editions not only for the group of novice participants as they point out [83].

However, while for Avourdiadou and Theodorakis [83] GS is a determining factor in IF, especially for experienced people, this study finds that for both novices and experienced people, the GS and PV dimensions on their own are not as relevant in terms of their effect on IF and that they need combinations between them or with GQ to reflect some type of explanatory capacity.

Similarly, it can be concluded that novice participants tend to rate the event more generally positively, as they are based on a less complex assessment scheme than experts [83]. They are more conformist with the production of the event, achieving more easily satisfaction with it, which influences their intention to participate in future editions. Furthermore, for this group of athletes, the quality–price ratio of the event is associated with their satisfaction with it (GS), which determines their willingness to participate in the future. On the other hand, for the experienced ones, due to their accumulated experience, the relationship between utility, pleasure and effort, and time and money invested are not enough determinants to decide or not to repeat the participation, so they focus more on more specific aspects, such as the work of the staff, the management of the specific elements of the event, or the communication of clear and precise information about the development of the competition.

## 6. Conclusions

Therefore, bearing in mind the effect of the variable experience on the perception of the quality of sports events and the intention of future participation, it is clear that sports managers need to design strategies individually for each segment.

On the one hand, as far as the more experienced athletes are concerned, care should be taken with aspects related to personnel, the specificity of the event, and communication in particular, all of which impact on overall quality and general satisfaction. The experienced participants, having more contrasted information, rely more on previous satisfaction judgments to be able to decide on future behavior, an aspect already pointed out by Bolton [110], and look at more specific details to determine whether the event is of a higher quality than others.

On the other hand, if we consider that the novice participants are less informed and will, therefore, value the competition more generically, we can think that to stimulate their intention to participate again, the managers should insist on the recreational nature of the event, generating positive emotions and good experiences.

In accordance with the above, the results of this study coincide in certain aspects with previous work. However, there is no unanimity on the variables that affect the future intentions of the participants, and in the case of the evaluation of experience, the research is very succinct. This situation can be a limitation because there are no repeated studies over time where the time series can be verified. Hence, it is considered necessary to go deeper into the topics addressed in this work, from a dynamic perspective, with the aim of shedding light on which aspects are indeed necessary for participants to value repeating the experience, differentiating the different perspectives of the experienced and novice participants.

Another limitation of this research refers to the fact that it is not possible to generalize the results, as the study was carried out with an incidental sample. Furthermore, it depicts a specific type of event: Duathlon, which, although it can be applied to similar studies on the triathlon modality, will require the adaptation of the questionnaire to other types of sporting events.

Nevertheless, results are considered useful to represent the phenomenon studied, taking into account the limited literature that addresses it from the participants’ perspective.

As future lines of research, considering the low female participation (ratio 1 to 3), it would be interesting to carry out studies with a gender perspective to find out whether some variables, such as “SD”, have a significant influence on future intentions to participate in this type of sample. Other possible lines of research would be to apply the study in different contexts, to analyze how it affects (in addition to the aforementioned gender) nationality, the type of competition, or the athletic goal achievement, and to introduce variables in the design of the instrument that evaluates the security protocols created as a result of the pandemic by the COVID-19.

## Figures and Tables

**Table 1:** Hypotheses testing.

Hypothesis	Relations (Path Coefficients)	Experienced β(t)	Novice β(t)	Experienced Test	Novice Test
H1a	S → GQ	0.2753 *** (3.0402)	0.3636 *** (4.1485)	Supported	Supported
H1b	C → GQ	0.1969 ** (2.3891)	−0.0535 (0.5960)	Supported	Not supported
H1c	CS → GQ	0.0803 (0.9522)	0.0569 (0.4203)	Not Supported	Not Supported
H1d	L → GQ	0.0965 (0.8605)	0.0504 (0.3707)	Not Supported	Not Supported
H1e	DS → GQ	0.3513 *** (3.8040)	0.5503 *** (3.3000)	Supported	Supported
H2	GQ → PV	−0.0051 (0.0214)	0.5685 *** (8.7856)	Not Supported	Supported
H3	GQ → GS	0.8427 *** (18.2806)	0.7469 *** (8.6920)	Supported	Supported
H4	PV → GS	−0.0075 (0.1102)	0.0625 (0.7200)	Not Supported	Not Supported
H5	PV → FI	0.0914 (1.1054)	0.1948 *** (3.4959)	Not Supported	Supported
H6	GS → FI	0.8044 *** (9.8137)	0.7607 *** (14.5086)	Supported	Supported

Notes: ** *p* < 0.01; *** *p* < 0.001. S: Staff; GQ: Global Quality; C: Communication; CS: Complementary Services; L: Logistics; DS: Duathlon Specifics; PV: Perceived Value; GS: General Satisfaction; FI: Future Intention.

**Table 2:** Sufficient conditions for experienced (E) and novice (N) participants.

Frequency Cut-Off: 2	Future Intentions (FI)					Future Intentions (~FI)			
Consistency Cut-Off	E = 0.826		N = 0.826			E = 0.808		N = 0.805	
	1	2	1	2	3	1	2	1	2
GQ	•		•	•		○		○	
PV		•	•		•		○		
GS		•		•	•		○		○
Raw coverage	0.843	0.644	0.707	0.743	0.640	0.828	0.679	0.795	0.824
Unique coverage	0.266	0.067	0.091	0.127	0.023	0.193	0.044	0.089	0.117
Consistency	0.819	0.880	0.895	0.892	0.923	0.851	0.912	0.835	0.863
Overall solution coverage		0.910			0.858		0.872		0.913
Overall solution consistency		0.807			0.848		0.820		0.784

Notes: • presence of condition; ○ absence of condition. GQ: Global Quality; PV: Perceived Value; GS: General Satisfaction.

## Data Availability

The raw data supporting the conclusions of this article will be made available by the authors, without undue reservation, to any qualified researcher.
